# Complicated appendiceal diverticulitis a extremely rare cause of acute abdomen: A case report and literature review

**DOI:** 10.1016/j.ijscr.2023.108535

**Published:** 2023-07-28

**Authors:** Giuseppe Evola, Giovambattista Caruso, Elia Pulvirenti, Maria D'Angelo, Martina Reina, Giuseppe Angelo Reina

**Affiliations:** a General and Emergency Surgery Department, Garibaldi Hospital, Piazza Santa Maria di Gesù 5, 95124 Catania, Italy; b General Surgery Department, Santissimo Salvatore Hospital (ASP Catania), Paternò, Catania, Italy

**Keywords:** Perforated appendiceal diverticulum, Appendicular diverticulitis, Acute appendicitis, Acute abdomen, Emergency surgery, Case report

## Abstract

**Introduction and importance:**

Appendiceal diverticulitis (AD) represents a rare cause of acute abdomen. Diagnosis of AD is a challenge because of its rarity and resemblance to other ileocecal diseases like as cecal diverticulitis (CD) and acute appendicitis (AA). Preoperative imaging can be useful to aid diagnosis. Surgery represents the correct treatment of AD.

**Case presentation:**

A 48-year-old Caucasian male presented to the Emergency Department with a two-day history of right lower quadrant (RLQ) abdominal pain and fever. Physical examination revealed RLQ abdominal pain and rebound tenderness with muscle guarding. Laboratory tests reported high levels of C-reactive protein and neutrophilic leukocytosis. Abdominal computed tomography(CT) scan showed findings of AA and a thin-walled 5 mm appendiceal diverticulum. The patient underwent laparoscopic appendectomy. The postoperative course was uneventful, the patient was discharged on the 5th postoperative day in a stable condition. Gross anatomy confirmed the presence of appendiceal diverticulum in the distal appendix on the mesenteric border. Histopathological examination revealed an inflamed and perforated appendiceal pseudo-diverticulum with surrounding AA and peri-appendicitis.

**Clinical discussion:**

Appendiceal diverticulosis is an uncommon entity, classified as congenital or acquired based on the number of appendiceal layers herniating through the normal wall. Two thirds of diverticula will develop acute or chronic diverticulitis that can lead to several complications some of which can be life-threatening.

**Conclusion:**

AD is a rare surgical emergency and represents often an overlooked diagnosis. Early diagnosis and treatment are crucial for reducing morbidity and mortality Appendectomy represents a safe and appropriate treatment of AD.

## Highlights

• Appendiceal diverticulitis (AD) represents a rare cause of acute abdomen.
• Diagnosis of AD is a challenge because of its rarity and resemblance to other ileocecal diseases.
• Preoperative imaging can be useful to aid diagnosis.
• Surgery represents the correct treatment of AD.

## 1 Introduction

Appendiceal diverticulosis is a rare disease. Appendiceal diverticula are classically divided into acquired and congenital types: the majority are acquired with an incidence of 0.004–2.1 % from appendicectomy studies and of 0.20–0.66 % from autopsy studies [1]. Appendiceal diverticula are often incidental findings diagnosed postoperatively through histopathological evaluation. Appendiceal diverticulitis (AD) represents a frequently underdiagnosed entity differing from acute appendicitis (AA) by the presence of an inflamed appendiceal diverticulum. Acquired appendiceal diverticulosis and diverticulitis usually affect older male adults. AD has an increased risk of perforation and a higher mortality rate compared to AA [[1], [2], [3], [4]]. A rare case of inflamed and perforated appendiceal diverticulum with surrounding AA is presented with review of the literature in accordance with SCARE 2020 criteria [5]. The purpose of this case report is to discuss that complicated AD is a rare cause of acute abdomen that requires emergency surgery.

## 2 Presentation of case

**Fig. 1:**
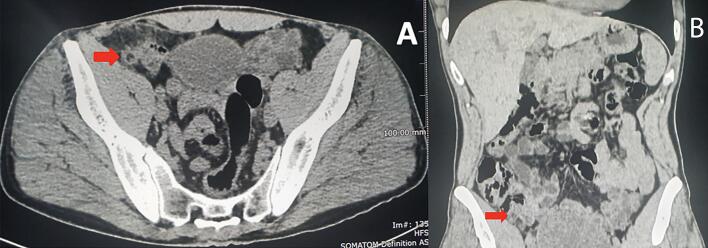
A,B. Abdominal CT showing a dilated appendix with thickened walls and a thin-walled 5 mm appendiceal diverticulum (red arrow). A transverse view, B coronal view. (For interpretation of the references to colour in this figure legend, the reader is referred to the web version of this article.)

**Fig. 2:**
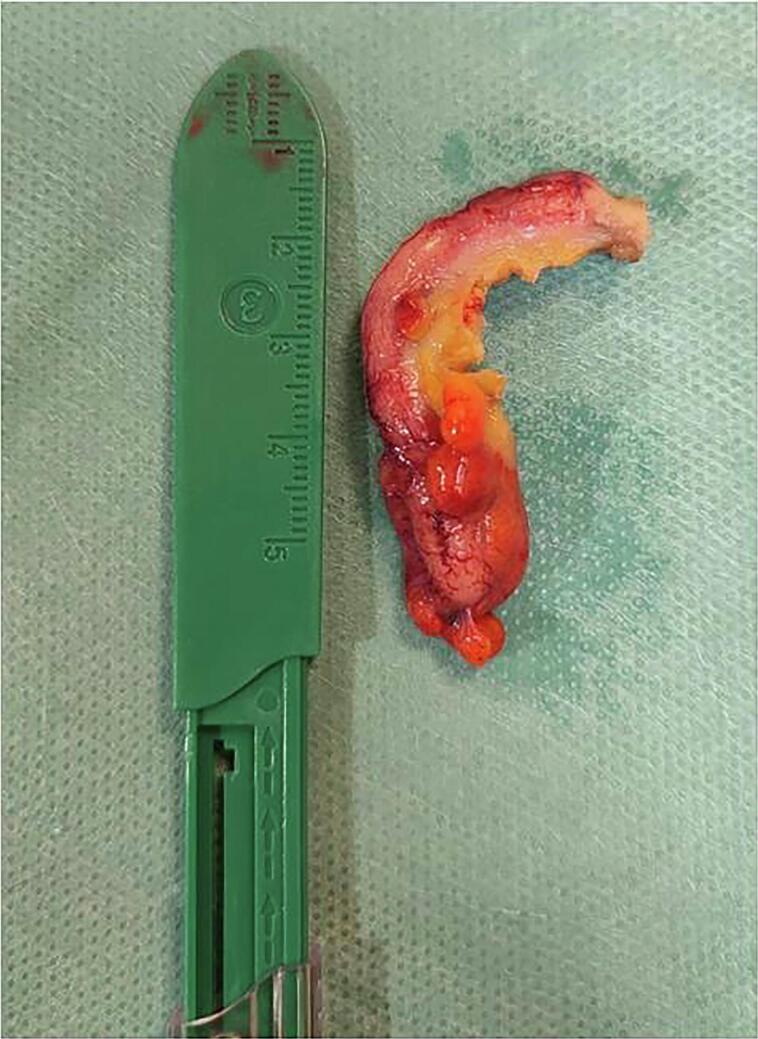
Acute appendicitis: operative findings.

**Fig. 3:**
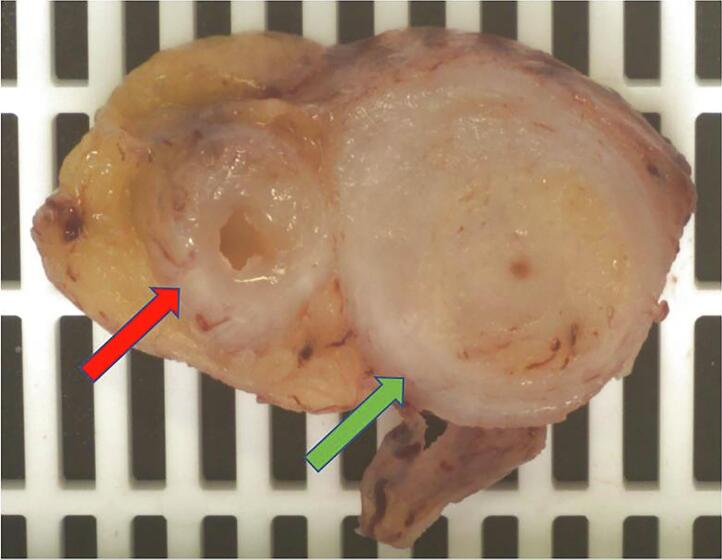
The surgical specimen showing a diverticulum (red arrow) in the distal third of the appendix on the mesenteric border and the appendiceal lumen (green arrow). (For interpretation of the references to colour in this figure legend, the reader is referred to the web version of this article.)

**Fig. 4:**
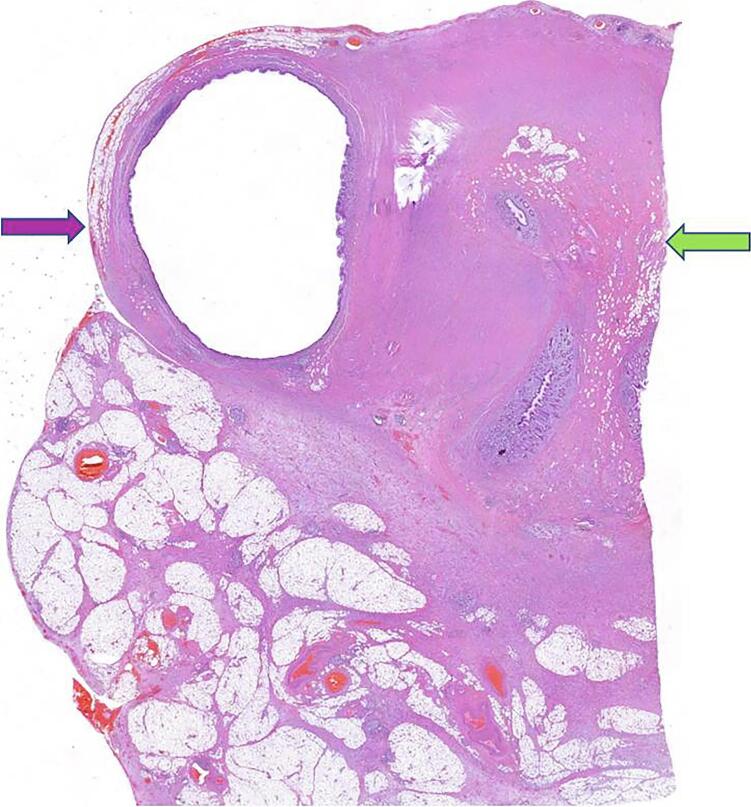
Photomicrograph section of appendiceal diverticulitis with surrounding acute appendicitis. Appendiceal diverticulum (purple arrow), appendiceal lumen (green arrow). Haematoxylin and eosin (original magnification x 40). (For interpretation of the references to colour in this figure legend, the reader is referred to the web version of this article.)

A 48-year-old Caucasian male presented to the Emergency Department with a two-day history of right lower quadrant (RLQ) abdominal pain and fever (38 °C); he had no nausea and vomiting, others vital signs were normal. The patient referred habit on smoking but denied alcohol consumption; his past and familial medical histories were normal. He was employed by profession, married and of medium socio-economic status. Physical examination revealed mild abdominal distention, RLQ abdominal pain with obvious muscle guarding and rebound tenderness, hypoactive bowel sound. Laboratory tests reported high levels of C-reactive protein (45.5 mg/L) and neutrophilic leukocytosis (WBC 16.700 10^3^/μL). The patient was managed with fluids, intravenous broad-spectrum antibiotics, antipyretic drugs and bowel rest. The patient, after a non diagnostic abdominal ultrasonography (US) due to the presence of large amounts of intestinal gas, was evaluated by abdominal computed tomography (CT) scan which revealed findings of AA like as the presence of a dilated appendix with thickened walls, a peri-appendiceal fat stranding and a pericecal fluid collection; a thin-walled 5 mm appendiceal diverticulum was also noted (Fig. 1A,B). The patient, after understanding the severity of his medical condition and accepting surgery, was taken emergently to the operating room by experienced general surgeons for laparoscopic appendectomy. After induction of pneumoperitoneum with the Veress needle and placement of three trocars (two 12-mm trocars in the umbilical region and in the left flank and one 5-mm trocar in the left iliac fossa) we explored the peritoneal cavity with evidence of AA and localized peritonitis. After dissection of mesoappendix, cauterization of appendicular artery with bipolar forceps and application of two endoscopic loop ties at the base of the appendix, the appendix was sectioned and removed in an endobag (Fig. 2). After drainage of pericecal abscess, a laminar pericecal drain was placed. Patient was given an IV injection of Amoxicillin/Clavulanate 2 g twice daily and Metronidazole 500 mg thrice daily for five days. The postoperative course was uneventful, laboratory tests were unremarkable and abdominal drain was removed on the 3th postoperative day. The patient was discharged on the 5th postoperative day in a stable condition. Gross anatomy confirmed the presence of diverticulum in the distal appendix on the mesenteric border (Fig. 3). Histopathological examination revealed an inflamed and perforated appendiceal pseudo-diverticulum (complicated AD) with surrounding AA and peri-appendicitis (Fig. 4). The patient tolered the advice provided (low fiber diet for 15 days) and after a follow-up of one month is asymptomatic.

## 3 Discussion

Appendiceal diverticulosis is an uncommon entity classified as congenital or acquired. Congenital diverticula (3 % of diverticula) are true diverticula because they contain all the layers of the appendiceal wall and tend to be single and located at the antimesenteric margin of the appendix. Acquired diverticula (the most common type) are pseudodiverticula (false) because only the mucosa, submucosa and serosa are the components of the diverticulum wall without the muscle layer [1,6]; they are often small (2–5 mm), multiple and located in the distal appendix on the mesenteric edge [3]. Congenital diverticula have been associated with chromosomal anomalies (Down syndrome, Patau syndrome) [3], appendiceal duplication, unsuccessful recanalization of the appendiceal lumen, adhesion traction to the appendix wall, remnant of epithelial inclusion cysts in the appendix, failed obliteration of the vitelline duct [1,7]. Hypotheses on the development of acquired diverticula advocate either inflammatory or noninflammatory causes. The inflammation hypothesis states that several episodes of inflammation or infection lead to atrophy of lymphoid tissues, resulting in a weaker and thinner residual wall. The noninflammation hypothesis holds that increased intraluminal pressure causes acquired diverticula to develop [1,7]. Risk factors associated with acquired diverticula are male sex, age older than 30 years, Hirschsprung's disease or cystic fibrosis. In the literature there aren't described any distinct risk factors that specifically contribute to the development of AD rather than in other location. Although appendiceal diverticulosis is usually silent, two thirds of appendiceal diverticula will develop acute or chronic diverticulitis that can lead to several complications some of which can be life-threatening. AD is generally caused by partial or complete obstruction of the appendiceal lumen. The most common complication of AD is perforation: acquired diverticula have a higher risk of perforation than that of congenital diverticula (up to 66 % vs 6.6 % respectively) due to a thin wall and lack of a thick muscularis propria. AD has a 3-fold higher risk of perforation and a 30-fold higher mortality risk compared to uncomplicated AA [7] and it is associated with a greater risk of neoplasms (carcinoids, mucinous adenomas, tubular adenomas, adenocarcinomas) [8]. Other complications of AD are gastrointestinal bleeding, abscess formation, pelvic pseudocyst formation, appendicovesical fistula formation [1]. Classification: appendiceal diverticulosis can be complicated by inflammation (AD) and morphologically classified into four types. Type 1 is the presence of acute diverticulitis with a normal appendix; type 2 the presence of acute diverticulitis with appendicitis; type 3 a noninflamed diverticulum with appendicitis; type 4 a noninflamed diverticulum with a normal appendix [9,10]. Lee et al. postulate that accompanying AA is often secondary to AD and that types 1 and 2 represent different stages in the progression of same disease process [11]. According to this classification, our patient had type 2 AD with ruptured AA. AD is usually overlooked because of its rarity and resemblance to other ileocecal diseases (CD and AA) [12,13]. Clinical presentation: although AD presents with similar symptoms and signs to AA [[14], [15], [16]], some differences have been observed. Compared to AA clinical presentation AD has a longer duration of pain (1–14 days), a greater presence of RLQ abdominal pain, a lower frequency of nausea and vomiting and it develops mainly in adults [1]. Diagnostics: abdominal US and CT scan can be useful to aid diagnosis [3,17], but their utility is highly radiologist-dependent. US findings of AD include a focal or diffusely thickened appendiceal wall (>3 mm), non-compressible firm dilated organ and well-defined hypoechoic round or oval-shaped cysts attached to the enlarged appendix, in the case of perforation the diverticulum wall will appear in discontinuity with presence of fluid collection [18]. CT scan is the imaging modality of choice by which the patterns of AD can be recognized [3]. However the use of CT scan is controversial because some authors reported that CT scan can overlook the appendiceal diverticulum because of its small size and cannot differentiate AD from cecal diverticulitis or AA [4], other authors believe that CT scan can be very useful because it has diagnosed AD in 86 % of cases [11]. CT findings of AD include visualization of inflamed diverticulum (small round cystic outpouching with contrast enhancement at the cystic wall) and appendicular thickening, absence of an appendicolith or fluid collection in the appendix and the formation of a phlegmon or an abscess. The sensitivity and specificity of CT for diagnosing AD is 48 % and 99 % respectively, this can be improved by increasing the effectiveness of CT image acquisition and reconstruction [19]. In our case report, although appendiceal diverticulum was evident on CT scan images, the features were consistent with AA and there was no evidence of AD. In practice, preoperative radiological diagnosis is frequently missed and AD is diagnosed intraoperatively or postoperatively at pathology if the diverticulum is small and located on the mesenteric border. Treatment: although laparoscopic appendectomy is the treatment of choice for symptomatic and complicated AD, recent literature discusses a role for prophylactic appendectomy when appendiceal diverticula are found incidentally due to the high risk of future AD and its complications as well an increased association with neoplasms [2,20]. Complications of laparoscopic appendectomy include bleeding (from the mesoappendix, omental vessels, or retroperitoneum), wound infection, trocar site hernias, visceral injury (bladder, small bowel, colon), incomplete appendectomy, intra-abdominal abscess, missed diagnosis (cecal neoplasms)**.** Pathological evaluation is required for the definitive diagnosis of AD, as in our case report, and to exclude concomitant neoplastic disease.

## 4 Conclusion

AD is a rare surgical emergency and represents often an overlooked diagnosis. The differential diagnosis of AD in patients presenting with signs and symptoms of AA is important as AD is associated with a higher risk of perforation and mortality in contrast to AA and it has also an increased risk of appendiceal neoplasms. Appendectomy represents a safe and appropriate treatment for symptomatic and complicated AD.

## Ethical approval

Ethical approval has been exempted by our institution because this is a case report and no new studies or new techniques were carried out.

## Sources of funding

This research did not receive any specific grant from funding agencies in the public, commercial, or not-for-profit sectors.

## CRediT authorship contribution statement

Giuseppe Evola: Drafting the manuscript, literature research.

Giovambattista Caruso: Drafting the manuscript, literature research.

Elia Pulvirenti: Operated on the patient, drafting the manuscript.

Maria D'Angelo: Drafting the manuscript, literature research.

Martina Reina: Drafting the manuscript, literature research.

Giuseppe Angelo Reina: Operated on the patient, revising the manuscript.

The guarantor for this case report is Giuseppe Evola.

## Guarantor

The guarantor for this case report is Giuseppe Evola.

## Consent

Written informed consent was obtained from the patient, for publication of this case report and accompanying images. A copy of the written consent is available for review by the Editor-in-Chief of this journal on request.

## Registration of research studies

Not applicable.

## Provenance and peer review

Not commissioned, externally peer-reviewed. The journal has a double anonymised peer review process.

## Declaration of competing interest

The authors have no conflict of interest to declare.
